# Interrelation of Oxidative Stress and Genetics in Pathophysiology of Obesity and Obesity-Related Conditions

**DOI:** 10.3390/genes16050489

**Published:** 2025-04-25

**Authors:** Emina Čolak, Lepša Žorić

**Affiliations:** 1 Institute of Medical Biochemistry, University Clinical Centre of Serbia, 11000 Belgrade, Serbia; 2 Clinic for Eye Diseases, University Clinical Centre of Serbia, 11000 Belgrade, Serbia; zoriclepsa@gmail.com; 3 Faculty of Medicine, University of Priština in Kosovska Mitrovica (UPKM), 40000 Kosovska Mitrovica, Serbia

**Keywords:** antioxidant enzymes, oxidative stress, obesity, SNPs, transcription factors

## Abstract

Obesity is a medical condition influenced by many factors and manifested by the excessive accumulation of fat. It is well documented that oxidative stress plays a significant role in the development of obesity and its related diseases. The antioxidant system’s enzymes, such as catalase, superoxide dismutase, glutathione peroxidase, paroxonase, etc., play a significant role in maintaining the oxidant–antioxidant balance in living organisms. Genetic variants of antioxidant system genes may affect the antioxidant system and its efficacy, which can lead to increased oxidative stress and higher risk for the development of obesity and its comorbidities. This review is focused on genetic variants such as single nucleotide polymorphisms of some antioxidant enzymes, ROS generators and transcription factors, and their impact on increased oxidative stress and the development of obesity and medical conditions related to obesity, like insulin resistance and metabolic syndrome.

## 1. Introduction

The prevalence of obesity has been constantly increasing for more than four decades in children, adolescents and adults worldwide. In 2016, it was estimated that almost 2 billion of adults were overweight (defined by body mass index—BMI), having a BMI ≥ 25 kg/m^2^, and 671 million were obese with a BMI over 30 kg/m^2^ [1].

It is well documented that oxidative stress, chronic inflammation and genetics are closely associated with obesity and its related conditions, such as insulin resistance, metabolic syndrome, diabetes type 2, cardiovascular diseases, etc. [2]. Increased oxidative stress is a result of an abnormal production of reactive oxygen species and/or a diminished antioxidant defense system.

It has been confirmed that genetic variations, known as single nucleotide polymorphisms (SNPs), may affect the functioning of the enzymes of the antioxidant system [3], even though the effect of genetic variations of genes associated with oxidative stress regulation has not been fully investigated in this particular medical condition. Further studies in this particular field are needed in order to acquire more clarity on mechanisms which are involved in the development of obesity and its comorbidities, such as insulin resistance and metabolic syndrome [4].

Increased concentration of free radicals such as superoxide anion, hydroxyl radical, hydrogen peroxide, etc., usually lead to the oxidation of biomolecules in the blood and cells. The enhanced oxidative stress associated with obesity often affects proteins, lipids and DNA, leading to the alteration of gene expression and modulation, and signaling pathways as well [5]. Antioxidant enzymes such as glutathione peroxide (GPX), superoxide dismutase (SOD), catalase (CAT), glutathione-S-transferase (GST) and others form the first line of antioxidant defense against the effect of free radicals and increased oxidative stress [2]. In fact, obesity is considered a low-grade chronic inflammation disease. Increased oxidative stress induces inflammation via adipogenesis stimulation [6]. Many enzymes involved in maintaining the balance between oxidative stress and antioxidant protection are damaged, with reduced or increased function, altered antioxidant capacity and changed oxidant–antioxidant balance due to various SNP mutations.

As a result, there is an excess of free radicals, increased oxidative stress and increased degree of chronic inflammation, which leads to an increased risk for the development of many diseases. Metabolic syndrome, obesity and insulin resistance are just some of them. In the continuation, we will provide an overview of the most important antioxidant enzymes, ROS synthesizers and transcription factors, the SNP mutations of which most often lead to obesity, metabolic syndrome and insulin resistance.

Recent findings suggest that transcription factors and epigenetic changes have a crucial role in metabolic-related gene regulation. Some authors suggest that transcription factors could serve as an attractive target for effective treatment of metabolic disorders such as obesity in the future [7].

The aim of this review is to summarize the current knowledge of the most known SNP variations of the antioxidant defense system genes, ROS generators and related transcription factors genes, and their interrelation with obesity and obesity-related conditions.

## 2. Material and Method

For this review, we used the literature that has been published on PubMed for the last 20 years. We included articles that aimed to investigate the role of SNP genes related to the risk of obesity and its metabolic complications. The gene selection was conducted according to their implication in redox processes and association with BMI, obesity, metabolic syndrome and insulin resistance. The selection criteria included the following: original articles published in English, conducted in humans, case-control, cross-sectional studies, randomized-control studies and meta-analyses that address obesity and its comorbidities, such as metabolic syndrome and insulin resistance.

## 3. Antioxidant Enzymes

### 3.1. Glutathione Peroxidase (GPx)

Several SNP variations associated with obesity and insulin resistance have been described so far, especially GPx1 and GPx7 genes. For example, the GPx1 gene has a missense polymorphism, which consists of the substitution of C to T at nucleotide 594, and which is manifested by the substitution of leucine for proline at codon 198 of the protein (*Pro198Leu*; *rs1050450*). Previous research has shown that male Leu allele (T) carriers have higher prevalence of metabolic syndrome, increased waist-to-hip ratio, triglycerides, HOMA-β (Homeostasis Assessment Model for pancreas functioning) and blood pressure as well, while women carriers had higher BMI, insulin and HOMA-IR (Homeostasis Assessment Model for insulin resistance) index (Table 1) [8]. Additionally, Leu allele carriers also have significantly higher levels of low-density lipoproteins (LDL), lipoperoxides and malondialdehyde (MDA) [9].

It was demonstrated that the combination of *Pro198Leu SNP* with the copy number variant (CNV) Ala5/Ala6 at codon 7–11 reduces the enzyme activity by 40% in in vitro conditions [9]. Hamanishi and assoc. showed that the combination of two other SNPs (-*602A/G*, *2C/T*) can decrease the transcriptional activity of GPX1 by 25% [10]. These data indicate that the Leu allele is associated with reduced GPX activity and thus, increased oxidative stress due to excess of ROS.

It was also shown that decreased GPx7 expression was found in several populations having SNP variations near this gene, and this condition was associated with obesity. The variant *rs835337 (G/A)*, located upstream of the gene, appeared to be positively associated with the body mass index (BMI). The minor allele (A) of this genetic variant was found to be associated with reduced BMI, increased GPX7 expression and decreased serum MDA concentrations. Thus, this variant provides protection against obesity by reducing the concentration of ROS, and therefore, it reduces the process of adipogenesis [4]. Finally, it can be concluded that these GPx SNP variants with enzymatic activity are involved in the protection against metabolic complications caused by obesity, while the sensor transducer GPx7 regulates the body’s response to elevated ROS synthesis in obesity.

### 3.2. Catalase (CAT)

Catalase, as one of the very significant antioxidant enzymes in the cell, degrades hydrogen peroxide (H_2_O_2_). It was found that this enzyme was substantially lower in obese children with insulin resistance [11,12]. Ruperez and assoc. found that some SNP variations of CAT located in the CAT promoter were significantly associated with obesity [11], especially the rare SNP variants *rs769214 (844A/G)*, *rs7943316 (89T/A)* and *rs1049982 (20C/T)*, which were significantly and positively associated with obesity in children. All these SNP variations were associated with reduced CAT expression and increased oxidative stress in human cell lines [13]. Another SNP variation, *SNP rs769214*, was found to be significantly associated with insulin resistance, obesity, higher BMI Z-score, adipocyte fatty acid-binding protein (A-FABP) and with higher plasma insulin concentration, without any effect on erythrocyte CAT activity as well [14]. Another studied variant was *SNP rs1001179 (262C/T)* in the 5′ untranslated region (UTR) of the CAT gene [15]. Some studies confirmed that the T allele was significantly associated with reduced CAT activity [16]. The presence of these SNPs could be manifested by lower CAT transcriptional activity and expression in the cells, leading to increased oxidative stress, impaired signaling cascades and increased dysfunction of macromolecules due to increased oxidation.

### 3.3. Superoxide Dismutase (SOD)

SOD acts as an important antioxidant enzyme that scavenges superoxide anion through oxidation/reduction cycles in the cells, in which superoxide anion is decomposed in hydrogen peroxide and molecular oxygen. These reactions take place in the presence of transition metals that are located in the active center of the enzyme [17].

Several studies demonstrated that EC-SOD levels were significantly increased in the adipose tissue and serum of obese mice along with IL-1β and TNFα as a consequence of increased oxidative stress response of adipose tissue [18]. Yang and assoc. demonstrated that Chinese patients with *Leu53Leu*, *Arg213Gly* and *Ala40Thr* polymorphisms in extracellular SOD (EC-SOD) had increased BMI, fasting insulin and higher susceptibility to type 2 diabetes mellitus [19].

Increased expression of SOD1/SOD2, together with reduced oxidative stress, was found in obese mice, suggesting to a protective effect of this enzyme against insulin resistance and induced glucose intolerance [20]. It was documented that SOD2 SNP rs-4880(C/T), located on the second exon, could generate change in the 16th protein amino acid from alanine to valine [21]. The Val-MnSOD variant is associated with reduced formation of Mn-SOD tetramer, and therefore, with a significant impairment of the process of superoxide anion dismutation [22]. It is considered that *ValVal* and *AlaAla* homozygous have an increased risk for various diseases due to increased concentration of oxygen radicals such as O_2_− and H_2_O_2_ [23]. It has been confirmed that people with the ValVal genotype have elevated concentrations of pro-inflammatory cytokines IL-1, IL-6, TNFα and interferon γ, and decreased concentrations of anti-inflammatory cytokines such as IL-10 [24]. Caple et al. showed that the Val allele carriers in healthy people had lower level of DNA damage [25].

All these findings indicate that the role of superoxide anion is very important in the development of obesity and its related disorders, as well as the activity of MnSOD as one of the main enzymes in the regulation of ROS concentration in cells. Maintaining the MnSOD activity within physiological limits would avoid possible complications and prevent the development of obesity-related diseases and metabolic complications.

### 3.4. Paraoxonase—PON

The Paroxonase family (PON) consists of three isoenzymes, PON1, PON2 and PON3, bound mainly to HDL-lipoproteins. Their function is to inhibit lipid peroxidation in HDL and LDL particles. So far, the study of Labreque et al. [26] found that only PON3 mRNA expression was positively associated with total body fat and body weight. The studies of Aslan [27], Ferretti [28] and Bajnok [29] showed decreased PON1 activity in obese individuals, while the studies of Martinez-Salazar [30], Veiga [31] and Tabur [32] showed no changes in PON1 activity in obese subjects. Adkins et assoc. [33] documented that subjects with the *192RR (ArgArg)* genotype have higher PON1 activity, as well as subjects with *55LL (LeuLeu)* that exhibit higher PON1 concentrations [34]. Another study of Veiga et al. [31] showed significant association of the R allele and obesity in Portuguese women. Barath et al. [35] found no correlation between PON1 SNP mutation and obesity development in adolescents. On the other hand, Ruperez and assoc. Ref. [36] found a novel PON1 *SNP rs854566* mutation that was inversely associated with obesity in children.

**Table 1:** Genetic variants of genes involved in oxidative stress and their consequences on obesity and obesity-related conditions.

**Gene**	**Genetic Variant**	**Consequences**	**References**
GPX1	rs105045	Leu allele T carriers have higher prevalence of MS, increase WHR, TG, HOMA-β, BMI, insulin, HOMA-IR, LDL-ch., lipoperoxides and MDA	[7,8]
	Pro198Leu		
	Pro198Leu;	Decreased GPX1 activity by 40%, increased oxidative stress, higher risk of obesity and its complications	[9]
	Ala5/Ala6 at codon 7–11		
	-602A/G, 2C/T	Decreased GPX activity by 25%, increased oxidative stress, higher risk of obesity and its complications	[9]

GPX7	rs835337 (G/A)	Increased BMI, decreased MDA, increased GPX7 activity in adipose tissue	[4]
CAT	rs 769214; -844A/G	Reduced CAT activity, increased oxidative stress, increased risk for obesity	[12,13]
	rs7943316; -89T/A;	Reduced CAT activity, increased oxidative stress, increased risk for obesity	
	rs1049982; -20C/T	Reduced CAT activity, increased oxidative stress, increased risk for obesity	
	rs1001179; -262C/T	Decreased CAT activity, increased obesity in children	[14]
EC-SOD	Leu53Leu	Increased BMI, fasting insulin, increased risk for type 2 diabetes mellitus	[18]
	ArG213Gly	Increased BMI, fasting insulin, increased risk for type 2 diabetes mellitus	[18]
	Ala40Thr	Increased BMI, fasting insulin, increased risk for type 2 diabetes mellitus	[18]
SOD2	rs4880 (C/T)	Increased obesity in elderly, lower enzyme activity, increased IL-1, IL-6, TNFα, and interferon activity	[20]
	(Ala16Val)		
	Val-MnSOD;	Increased oxidative stress due to decreased rate of superoxid anion dismutation, increased activity of pro-inflammatory cytokines and decreased activity of anti-inflammatory cytokines	[21]
	ValVal; AlaAla		[22,23]

PON1	rs854566	Increased obesity in children	[35]
	rs662 (Q192R) (G/A)	192RR, ArgArg; increased PON1 activity	[32]
		R-allele; increased risk for obesity	[30]
	rs854560 (L55M) (A/T)	Increased PON1 activity	[33]
PRDX3	rs3740562 (A/G)	Increased BMI in Japanese subjects on high-fat diet	[36,37,38]
	rs2271362 (C/T)	Increased BMI in Japanese subjects on high-fat diet	
	rs7768 (G/T); rs3377 (A/C)	Increased BMI in Japanese subjects on high-fat diet	
NADPH oxidase	rs9932581 (-930A/G)	Higher NADPH oxidase activity, increased ROS production, increased oxidative stress, insulin resistance and HOMA-IR	[39,40]
p22 phox	rs4673 (242 C/T)	Reduced concentration of 8.OhdG, lower HOMA-IR and fasting insulin in T allele type 2 diabetic carriers.	[41]
		Increased risk for obesity and type 2 diabetes mellitus in Brazilian hypertensive subjects	[42]
PPAR-γ	rs1801282 (Pro12Ala)	Reduced BMI, increased insulin sensitivity and decreased PPAR-γ transcriprion activity	[43]
PGC1α	rs8192678 (Gly482Ser)	Positive association with HOMA-IR, WHR, hyperglycaemia and TBARS, lower adiponectin levels in type 2 diabetic subjects	[44,45,46]
			[47]
NRF2	rs6721961 (-617C/A)	Lower NFR2 transcription activity	[48,49,50]
	rs6706649 (-651G/A)	Lower NFR2 transcription activity	
	rs6721961; rs4880	Reduced antioxidant defense system, increased risk for several diseases	[49]
	rs2234694	Affected SOD1 activity, increased risk of obesity	
	rs2536512	Affected SOD3 activity, increased risk of obesity	
	rs1056806	Affected GSTM1 activity, increased risk of obesity	
	rs1800668	Affected GPX1 activity, increased risk of obesity	[51]
	rs4880	Affected SOD2 activity, increased risk of obesity, increased concentration of ox-LDL-cholesterol, increased prevalence of type 2DM and its cardiovascular complications, and glucose intolerance	[52,53,54,55,56]


	rs1050450	Increased risk of obesity in Mexican female population	[54]
	rs6721961	Increased risk for development of type 2 DM in Mexican population	[57]
	rs2364723	Association with obesity and type 2 diabetes mellitus and diabetes-related complications	
	rs10497511	Association with obesity and type 2 diabetes mellitus and diabetes-related complications	
	rs1962142	Association with obesity and type 2 diabetes mellitus and diabetes-related complications	
	rs6726395	Association with obesity and type 2 diabetes mellitus and diabetes-related complications	[58,59]

Legend: MS—metabolic syndrome; WHR—waist-to-hip ratio; TG—triglycerides; BP—blood pressure; BMI—body mass index; HOMA-β—Homeostasis Assessment Model for pancreas functioning (β-cells); HOMA-IR—Homeostasis Assessment Model for insulin resistance (IR); T2DM—type 2 diabetes mellitus; LDL-chol.—low-density lipoprotein-cholesterol; ox-LDL—oxidized LDL cholesterol fraction; ROS—reactive oxygen species; 8-OHdG—8-hydroxy-2-deoxyguanosine; TBARS—thyobarbituric acid reactive substances; MDA—malondialdehyde; IL-1—Interleukin-1; IL-6—Interleukin 6; TNFα—Tumor necrosis factor α.

### 3.5. Thioredoxins/Peroxiredoxins—PRDX

Thioredoxins are a family of peroxidases that degrade hydrogen peroxide in the cells. The family consists of six isoforms that participate in ROS signaling, processes of cell proliferation and apoptosis. It was documented that PRDX3 could scavenge almost 90% of the hydrogen peroxide from mitochondria. Therefore, this isoenzyme is considered very important in the regulation of cell redox status [37]. The activity of PRDX3 was significantly decreased in the adipose tissue of obese subjects [38]. It was found that four PRDX3 SNP mutations (the *SNPs rs 40562 (A/G)*, *rs 2271362 (C/T)*, *rs 7768 (G/T)* and *rs 3377 (A/C)*) were associated with obesity and higher BMI in Japanese people who were fed with high-fat diet [38]. Hiroi and assoc. [60] found that the interaction of genotype and halotype with high-fat diet leads to obesity and increased BMI. Nevertheless, more studies are needed in order to elucidate the role of these mutations in increased risk of obesity and its metabolic complications.

## 4. ROS Producers

### NADPH Oxidase

The NADPH oxidase complex is the most important ROS producer in the course of phagocyte respiratory burst [39]. Insulin has a stimulating effect on this complex. In cooperation with cytokines, insulin stimulates the synthesis of ROS, which subsequently participate in the processes of signal transduction [40]. The NADPH complex consists of six units: p22phox and gp 91phox, which form cytokine b559, p47 phox, p67 phox, p40 phox and rac.

So far, it has been established that *SNP -930A/G* in the p22 phox promoter is associated with higher NADPH oxidase activity in phagocytic cells of hypertensive patients carrying the GC genotype [40]. A higher NADPH oxidase activity leads to higher ROS production and therefore, a higher risk of insulin resistance [61]. It was also documented that GC genotype was significantly positively correlated with HOMA-IR and insulin activity in obese Spanish subjects [41]. Hayaishi-Okano and assoc. [42] showed that the presence of *SNP 242 C/T* in the p22 phox type 2 diabetic Japanese carriers of T allele had significantly lower intima media thickness and reduced concentration of 8-hydroxy-2′-deoxyguanosine (8-OHdG). In the same article, the authors showed that the non-diabetic T allele carriers had lower HOMA-IR and fasting insulin values and an absence of insulin resistance [42]. The study of Schreiber et al. showed that *C242T* polymorphism was associated with obesity and diabetes mellitus of Brazilian hypertensive patients [62]. According to these findings, it can be concluded that NADPH oxidase is a very important modulator of the insulin signaling pathway, and represents a significant link between insulin resistance and obesity.

## 5. Transcription Factors

### 5.1. Peroxisome Proliferator-Activated Receptors—PPAR-γ

Peroxisome proliferator-activated receptors—PPAR—consist of three subunits, PPAR-α, PPAR-β and PPAR-γ, and form a superfamily of nuclear receptors. This superfamily is very important in the regulation of lipid and energy metabolism, mitochondrial biogenesis and antioxidant defense [63]. Itoh and assoc. showed that oxidative stress could affect the intracellular signaling through PPAR-γ, suggesting that PPAR-γ expression could be downregulated by ROS (hydrogen peroxide especially), TNF-α and lysophosphatidyl-choline [43]. The well-known SNP of PPAR-γ gene is *rs1801282*, responsible for changing the amino acid sequence at codon 12 from proline to alanine *(Pro12Ala).* The presence of Ala allele was associated with reduced BMI, increased insulin sensitivity and decreased transcription activity [64]. In another study of Galbete et al., a meta-analysis that included almost 50,000 subjects demonstrated quite the opposite: individuals who had the *Pro 12 Ala* variant of the PPAR-γ2 gene had higher BMI [65]. In any case, more studies are needed to elucidate the real function of this regulator.

### 5.2. Peroxisome Proliferator-Activated Receptor γ Coactivator 1-α—PGC1α

PGC1α is a very important regulator of mitochondrial biogenesis and liver glyconeogenesis. It is also involved in oxidative metabolism and development of oxidative stress [43,44]. The SNP variation of PGC1α, *rs8192678*, results in amino-acid substitution of glycine to serine at position *482 (Gly482Ser)* [45]. The Gly482Ser variant was found to be positively associated with HOMA-IR [46], waist-to-hip ratio, TBARS concentration and hyperglycaemia [47]. Okauchi et al. found that the *Gly482Ser* variant was associated with lower adiponectin levels in type 2 diabetic males [66]. All these studies suggested that the *Gly482Ser* variant had a positive effect on obesity-associated comorbidities: insulin resistance and type 2 diabetes mellitus [66].

### 5.3. Nuclear Factor Erythroid 2-Related Factor—NRF2

NRF2 regulates the cellular defense against toxic and oxidative damage through the expression of genes involved in oxidative stress response and drug detoxification [67].

Bound to Kelch-like ECH-associated protein 1 (KEAP1) in the cytoplasm, NRF2 has been involved in proteosomal degradation. This transcription factor has been involved in the redox cycling of several redox enzymes, such as thioredoxin, thioredoxin reductase, sulfiredoxin, peroxiredoxin, glutathione peroxidase, superoxide dismutase 1, catalase and several glutathione S-transferase [48].

Using genome-wide association studies (GWAS), several sequence mutations of the NRF2 locus were found. It was documented that *SNP-rs6721961 (-617C/A)* and *rs6706649 (-651G/A)* in the promoter region of NRF2 will downregulate its transcription activity [49,50,68]. It was also found that SNPs *rs6721961* and *rs4880* of the NRF gene will lead to reduced antioxidant defense system and increased susceptibility to several diseases [50]. The polymorphism -*653A/G (rs35652124)* of NRF2 gene was found to be associated with increased liver inflammation in patients with alcoholic liver disease [51].

Several SNPs of the NRF2 gene have repercussions on certain antioxidant enzymes, for example, *rs2234694* is related to SOD1, *rs2536512* to SOD3, *rs1056806* to GSTM1, *rs4880* to SOD2 and *rs1800668* to GPx1; all were found in obese patients and were associated with increased risk of obesity [52]. It was also found that SNP *rs4880* was associated with increased concentration of oxidized LDL-cholesterol fraction (ox-LDL) and increased prevalence of type 2 diabetes [53], and glucose intolerance [54]. In addition, according to Gottlieb and assoc., the *rs4880* carriers have also increased risk for cardiovascular disease in patients with T2DM (type 2 diabetes mellitus) and inflammation related to ox-LDL fraction and higher progression of atherosclerosis [53]. Hernandez Guerrero and assoc. suggested that GPx *rs1050450* was associated with obesity development in the Mexican population, especially in females [55]. It was documented that this type of polymorphism was related to diabetic retinopathy in Polish and English populations [56,57], and with the development of carotid plaque in the Chinese population having diabetes [69]. According to Jimenez-Osorio et al., the polymorphism *rs6721961* of the promoter region of the NRF2 gene was associated with the development of T2DM in Mexican patients [70]. Wang et al. found that the same polymorphism was related to diabetic nephropathy in Chinese patients [58]. Finally, it was suggested that NRF2 gene polymorphisms *rs2364723*, *rs10497511*, *rs1962142* and *rs6726395* were found to be associated with obesity, diabetes type 2 and diabetes-related complications [59,71].

## 6. Discussion and Future Perspectives

The research that has been made in the last few decades points to the impact of increased oxidative stress on genetic regulation, proteins activities, and genome stability [72]. The genes that are involved in the regulation of oxidative stress such as generators of reactive oxygen species and their scavengers as well, (NADPH, CAT, SOD, GPX etc.) could be involved in the modulation of BMI, obesity and obesity-related comorbidities [52]. The interrelation between oxidative stress levels and genetic variations of several genes may be very important in regulation of BMI and obesity. In any case, this aspect should be further investigated. According to these facts, several genes that encode the synthesis of some antioxidant enzymes, ROS generators and transcription factors and are associated with obesity, metabolic syndrome, and insulin resistance were selected. A large and significant interrelation of oxidative stress with gene regulation was documented, as well as the influence of genes on the synthesis of various proteins. This confirms the great significance of oxidative stress interaction to genome regulation and genetic effects. Certain interactions failed to identify this significant effect, most likely due to small size and probably due to inherent variability of the populations. It should be taken into account that diverse SNPs are associated with several metabolic disorders and it is necessary to extend the research on each population, including and ethnic groups because each population has a specific set of genes. They are formed as a consequence of “gene-imprinting” and “gene-transfer”, lifestyle, diet, geographical area of residence, etc. 

In the Hortega Study, Lara-Hernandez et al. [71] studied the potential association between 723 SNPs located within a set of 212 genes. A group of 1502 Spanish adults were examined in order to elucidate the potential impact of these SNPs variations and oxidative stress levels on BMI. The selected genes were included in the regulatation of several biological processes, including obesity, blood pressure, inflammation, lipid metabolism and redox homeostasis. Their findings revealed a significant association between specific genes and both BMI and oxidative stress parameters. These authors observed that several SNPs were involved in the final physiological response in subjects under dietary interventions. They suggested that the SNPs -21A/T CAT (rs7943316) and 47C/T SOD_2_ (rs4880) reported in one of their previous article, could be used in future as a genetic tool to improve the treatment of overweight and obesity, as well as to evaluate the risk of developing several comorbidities in obesity [73]. The characterization of SNPs in obese subjects could contribute, also, to the development of controlled antioxidant therapies that could be potentially used for the treatment and prevention of obesity and its comorbidities. 

Most of the genetic variants associated with obesity and its comorbidities were identified through GWAS studies (genome-wide association studies) that were mostly conducted in European, Asian and partly in Latin-American population [74]. It was noticed that many common genetic variants in Europeans have not been identified in Asian population, due to differences in lifestyle habits, biological traits, cultural inheritance. Therefore, it should be emphasized that, genetic variants vary, depending on the origin, meaning that the population within the same continental groups, follow the same allelic patterns compared to intercontinental populations that showed different patterns [74]. Therefore, the SNPs genes identification that are involved in the pathogenesis of obesity across different populations could have a great impact on the diagnosis, treatment and prevention of disease across different populations.

In order to address this issue, and with the goal of better understanding the effect of oxidative stress on genomic variation and its effects on obesity and obesity-related conditions, the future studies must consider a larger sample sizes and analysis of the entire human genome.

## 7. Conclusions

Studies of genetic variants of antioxidant enzymes, as well as other genes that are involved in the synthesis and regulation of reactive oxygen species and transcription factors, are undoubtedly very important for a better understanding of the role of the antioxidant protection system in preventing obesity and its complications.

This review aimed to show in what way the antioxidant protection pathways, the processes of synthesis of free radicals, transcription factors, as well as factors that regulate signal transduction pathways are interconnected in obesity, and how genetic variations can lead to altered/reduced functions of certain enzymes and the development of obesity and other obesity-related disorders. Additionally, more studies are needed in the future to clarify this interconnection.
